# Changes in Depression and Stress after Release from a Tobacco-Free Prison in the United States

**DOI:** 10.3390/ijerph13010114

**Published:** 2016-01-12

**Authors:** Jacob J. van den Berg, Mary B. Roberts, Beth C. Bock, Rosemarie A. Martin, L.A.R. Stein, Donna R. Parker, Arthur R. McGovern, Sarah Hart Shuford, Jennifer G. Clarke

**Affiliations:** 1 Division of Infectious Diseases, The Miriam Hospital, 164 Summit Avenue, Providence, RI 02906, USA; 2 Center for Primary Care and Prevention, Memorial Hospital of Rhode Island, 111 Brewster Street, Pawtucket, RI 02860, USA; Mary_Roberts@mhri.org (M.B.R.); Donna_Parker@brown.edu (D.R.P.); Jennifer_Clarke@brown.edu (J.G.C.); 3 Centers for Behavioral & Preventive Medicine, The Miriam Hospital, Coro West, Suite 309, 164 Summit Avenue, Providence, RI 02906, USA; Beth_Bock@brown.edu; 4 Department of Behavioral and Social Sciences, Brown University School of Public Health, Box G-S121-4, Providence, RI 02912, USA; Rosemarie_Martin@brown.edu; 5 Department of Psychology, University of Rhode Island, Chafee Hall, 142 Flagg Road, Kingston, RI 02881, USA; LARStein@uri.edu; 6 Department of Psychology, Nichols College, 129 Center Road, Dudley, MA 01571, USA; Arthur.McGovern@nichols.edu; 7 Brown University School of Public Health, 121 South Main Street, Providence, RI 02912, USA; Sarah_Shuford@brown.edu

**Keywords:** depression, stress, prisoners, post-release, United States

## Abstract

Prior research has found high levels of depression and stress among persons who are incarcerated in the United States (U.S.). However, little is known about changes in depression and stress levels among inmates post-incarceration. The aim of this study was to examine changes in levels of depression and stress during and after incarceration in a tobacco-free facility. Questionnaires that included valid and reliable measures of depression and stress were completed by 208 male and female inmates approximately eight weeks before and three weeks after release from a northeastern U.S. prison. Although most inmates improved after prison, 30.8% had a worsening in levels of depression between baseline and the three-week follow-up. In addition, 29.8% had a worsening in levels of stress after release than during incarceration. While it is not surprising that the majority of inmates reported lower levels of depression and stress post-incarceration, a sizable minority had an increase in symptoms, suggesting that environmental stressors may be worse in the community than in prison for some inmates. Further research is needed to address depression and stress levels during and after incarceration in order for inmates to have a healthier transition back into the community and to prevent repeat incarcerations.

## 1. Introduction

In the United States (U.S.), over 2.2 million people are currently incarcerated [1]. Increasingly, those with mental health problems are disproportionately represented in prisons and jails in America [2]. Each year, approximately 700,000 persons (approximately 32%) with mental illness are admitted to U.S. correctional facilities. In addition, an estimated 56% of state prisoners, 45% of federal prisoners, and 64% of jail inmates in the U.S. reported having a recent history or symptoms of a mental health problem that occurred in the past year [3]. Indeed, it has been noted that correctional facilities currently house more persons with severe mental illness than in state hospitals throughout the country [4].

Depression and stress are particularly common among persons who are incarcerated in the U.S. [3]. Depression is characterized by “sadness, loss of interest or pleasure, feelings of guilt or low self-worth, disturbed sleep or appetite, feelings of tiredness, and poor concentration that is long-lasting or recurrent” [5]. In contrast, stress is defined as “a particular relationship between the person and the environment that is appraised by the person as taxing or exceeding his or her resources and endangering his or her well-being” [6]. The psychological effects of imprisonment and its implications for post-release contribute to inmates’ experience of both depression and stress during and after incarceration [7].

While much research indicates that risk for depression is influenced by both genetics and environmental factors, trauma, difficult life circumstances, or any stressful situation, such as incarceration, may trigger a major depressive episode [8]. For example, researchers in one study found that more than 40% of people recruited from a hospital’s emergency room in Israel with post-traumatic stress disorder also had depression four months after the traumatic event [9]. In the general population, there is also increasing evidence that chronic stressful life events coupled with poor coping strategies can increase the risk of developing depression and physical illnesses [10,11,12].

Little is known about changes in levels of depression and stress among inmates during incarceration and post-release. Examining changes in depression and stress levels among inmates during and after incarceration is particularly important as it could help us understand how to better assist inmates during the post-release reintegration process and to prevent repeat incarcerations. The current study focuses on investigating changes in depression and stress levels as inmates are released from prison and return back to the community. We hypothesized that depression and stress levels among inmates would mostly improve post-incarceration but that because of challenges associated with release to the community (e.g., obtaining housing, reestablishing connection with family and friends, securing income) some individuals may experience a worsening of symptoms. We were also interested in identifying what factors might impact differences in depression and stress levels according to specific demographic characteristics, including age, gender, race/ethnicity, education, annual income (prior to incarceration), length of incarceration, and living situation.

## 2. Methods

### 2.1. Data Collection

Data were drawn from the randomized controlled trial (RCT), “Project WISE” (Working Inside for Smoking Elimination), that investigated a smoking abstinence intervention conducted in a tobacco-free prison (cigarette smoking and the use of other tobacco products are not permitted in or around premises by inmates or staff) located in the northeastern U.S. [13,14]. The RCT evaluated the effectiveness of a six-session integrated cognitive-behavioral therapy and motivational interviewing intervention in comparison to a general wellness control condition that included viewing health videos that did not include mental health as a topic of discussion. The frequency and duration of participant contact between both conditions were equivalent. The RCT was reviewed and approved by the Memorial Hospital of Rhode Island Institutional Review Board and the Medical Research Advisory Group at the Rhode Island Department of Corrections (Office for Human Research Protection Federal Wide Assurance# for Memorial Hospital of Rhode Island is 00000346). In addition, a Certificate of Confidentiality was obtained to further ensure participant privacy.

Inclusion criteria for Project WISE required that inmates had to be ≥18 years old, report smoking ten or more cigarettes per day prior to incarceration, be able to communicate in English, and be scheduled for release from prison within eight weeks of study enrollment. Exclusion criteria were inability to give informed consent or housed in a segregation unit as there was limited access to these particular inmates due to their current status. Symptoms of depression resulting from cigarette smoking cessation were not a concern as the average length of time since their last cigarette was seven months, suggesting that withdrawal symptoms due to quitting cigarette smoking would have likely subsided by that point for the majority of inmates who participated in the current study. All eligible inmates who were interested in participating reviewed and signed the informed consent document administered by trained research staff.

Of the 312 inmates screened during the RCT, 273 met study eligibility and 262 agreed to participate and were consented. Baseline data (*n* = 247) were excluded from a total of 19 participants (nine whose data were lost due to a computer program error, six who became ineligible for the study after the baseline because they were never released from prison, and four who had missing data on the 10-item Center for Epidemiologic Studies Depression Scale (CES-D-10) that measures self-reported depression [15,16].) At the three-week follow-up (*n* = 220) an additional 12 participants were excluded due to a significant amount of missing data on the CES-D-10 and/or the 10-item Perceived Stress Scale (PSS) the measures self-reported stress [17]. The final total sample for the current study was 208, representing a response rate of 84.2% of the baseline sample.

### 2.2. Variables

Participants completed a 60-min Audio Computer-Assisted Self-Interview at baseline pre-release and at three weeks post-release.

*Depression* levels were assessed using the CES-D-10 [15]. Participants indicated how often in the past week they experienced specific feelings or behaviors on a 4-point Likert-type scale (0 = *rarely or none of the time* (less than one day) to 3 = *all of the time* (5–7 days)). Sample items include: “I felt depressed,” “My sleep was restless,” and “I felt lonely.” Specific items are reversed scored and all items are summed to provide an overall score ranging from 0 to 30 with higher scores indicating greater levels of depression. A score of 0–9 is “no depression”, a score of 10–14 is “mild depression”, a score of 15 or greater is “severe depression.” Internal consistency (Cronbach’s alpha = 0.86) and test-retest reliability for the CES-D-10 has been reported to be excellent [16]. In the current study, Cronbach’s alpha for the CES-D-10 was 0.84.

*Stress* levels were measured using the PSS [17]. Items, such as “In the last month, how often have you been upset because of something that happened unexpectedly?”, were rated by participants to indicate how often they felt or thought a certain way using a five-point Likert-type scale ranging from 0 (*never*) to 4 (*very often*). Specific items are reversed scored and item ratings are averaged with higher scores indicative of greater levels of perceived stress. A score of 0–13 is “no stress”, a score of 14–19 is “mild stress”, and a score of 20 or greater is “severe stress.” The total score Cronbach’s alpha for the PSS was 0.81 in this study.

*Demographic characteristics* included age, gender, race/ethnicity, education, annual income (prior to incarceration), length of incarceration, and living situation. A stable living situation was defined as living with a sexual partner, children, parents, other family, alone or with friends at the participant’s residence. An unstable living situation was defined as living with friends at the friend’s residence, shelter, on the street, or in a controlled environment.

*Perceived physical health* was assessed by asking participants to indicate how healthy they considered themselves to be rated as “excellent”, “very good”, “good”, or “poor.”

### 2.3. Data Analysis

IBM SPSS Statistics for Windows (version 20) was used to perform all statistical analyses. Demographic characteristics were calculated using descriptive statistics including frequencies, means, and standard deviations. Change in depression and stress were calculated for each scale separately by subtracting the baseline score from the three-week follow-up score. Positive difference values indicated worsened depression or stress and negative difference values indicated improved depression or stress. Each participant was coded as either improved or worsened for depression or stress. We compared those with improved *vs.* worsened depression and stress levels on demographic characteristics including age, gender (coded Male *vs.* Female), race/ethnicity (coded White, non-Hispanic; Hispanic; Black, non-Hispanic; and Other, non-Hispanic), education (coded <High School *vs.* High School or greater), annual income (coded <$20,000/year), length of incarceration (coded 6 months or less *vs.* more than 6 months), and living situation (coded stable *vs.* unstable) with chi-square analyses for categorical variables and independent t-tests for continuous variables. We conducted analyses to determine if there were differences between those participants who worsened and those participants who improved with respect to depression and stress levels from baseline to three-week follow-up. We hypothesized that the majority of participants would improve in stress and depression after release from incarceration, but that some participants may have an exacerbation of symptoms due to release to the community.

## 3. Results

### 3.1. Excluded/Included Participants

Excluded participants were those lost at three-week follow-up (*n* = 27) and those who were missing a portion of the assessment that included CES-D-10 and/or PSS (*n* = 12). No significant differences were found between those participants included (*n* = 208, 84.2%) and those excluded (*n* = 39, 15.8%) in age, race, education, and living environment. In comparison to excluded participants, participants included in the analyses had a higher monthly income prior to incarceration (*M* = $450, *SD* = $684 *vs. M* = $1753, *SD* = $4904, t(213) = −3.43, *p* = 0.001) and had a higher percentage who were incarcerated longer than six months (34% *vs.* 55%, χ^2^ = 5.74, df = 1, *p* = 0.02). None of the demographic characteristics were significantly related to change in CES-D-10 or PSS scores between baseline and three-week follow-up.

### 3.2. Descriptive Analysis for Final Sample

The majority of the final sample (*n* = 208) was 66.8% (*n* = 139) men with a mean age of 35.4 years (*SD* = 9.2). Fifty point nine percent (*n* = 106) self-identified as White, 18.3% (*n* = 38) as Black, and 30.7% (*n* = 66) as Multiracial/Other. In addition, 20.2% (*n* = 42) self-identified as Hispanic. Sixty-four point nine percent (*n* = 135) also had less than 12 years of formal education. The median annual income $7200 (inter-quartile range (IQR) = $15,600) and length of incarceration was 7.5 months (IQR = 18 months). Seventy-one point six (*n* = 149) percent of the sample reported living in a stable living environment prior to incarceration.

### 3.3. Depression and Stress Levels at Baseline and Three-Week Follow-Up

At baseline, 36.1% (*n* = 75) of participants had no depression (CES-D-10 score 0–9), 31.3% (*n* = 65) had mild depression (CES-D-10 score 10–14), and 32.7% (*n* = 68) had severe depression (CES-D-10 score of 15 or greater). Three weeks after release from prison, 58.2% (*n* = 121) had no depression, 21.6% (*n* = 45) had mild depression, and 20.2% (*n* = 42) had severe depression. The average baseline, three-week follow-up, and difference between baseline and follow-up CES-D-10 scores were 12.4 (*SD* = 6.3), 9.1 (*SD* = 5.9), and 3.3 (*SD* = 6.8), respectively. While 69.2% (*n* = 144) of participants improved, 30.8% (*n* = 64) had a worsening in their CES-D-10 score between baseline and the three-week follow-up.

At baseline, 31.7% (*n* = 66) of participants had no stress or mild stress (PSS score 0–19) and 68.3% (*n* = 142) had severe stress (PSS score 20–40). Three weeks after release from prison, 57.7% (*n* = 120) had mild or no stress and 42.3% (*n* = 88) had severe stress. The average baseline, three-week follow-up, and difference between baseline and follow-up PSS scores were 21.9 (*SD* = 6.2), 17.6 (*SD* = 5.6), and 4.3 (*SD* = 7.4), respectively. While 70.2% (*n* = 146) of participants’ stress improved, 29.8% (*n* = 62) had worse PSS scores after release than before release. The percentage of participants who had both depression and stress at baseline and three-week follow-up was 55.3% (*n* = 115) and 32.7% (*n* = 68), respectively.

### 3.4. Differences between Those Who Improved vs. Those Who Worsened in Depression and Stress

Significant differences were found between those participants who worsened and those who improved in depression and stress scores from baseline to three-week follow-up. Table 1 presents the group differences based on depression scores (improved *vs.* worsened) and Table 2 presents the group differences based on stress scores (improved *vs.* worsened). In comparison to those participants who improved, those who worsened in depression at the three-week follow-up were more likely to report an annual income of $20,000 or less (92.7%, *n* = 76 *vs.* 76.1%, *n* = 89) and identify their physical health status as poor/fair/good *vs.* very good/excellent (56.4%, *n* = 22 *vs.* 43.6%, *n* = 17). In addition, participants who worsened in depression at the three-week follow-up were more likely to report higher perceived stress (*M* = 21.36, *SD* = 4.27 *vs. M* = 14.88, *SD* = 4.91) and more likely to report severe stress (78.2%, *n* = 68 *vs.* 16.5%, *n* = 20). In comparison to those participants who improved, those who worsened in stress scores at the three-week follow-up were more likely to report living in an unstable living environment (32.1%, *n* = 27 *vs.* 17.9%, *n* = 20). Furthermore, participants who worsened in stress scores at three-week follow-up were more likely to report higher depression (*M* = 13.49, *SD* = 4.91; *M* = 5.82, *SD* = 4.30) and were more likely to report severe depression (39.8%, *n* = 35 *vs.* 5.8%, *n* = 7).

**Table 1:** Descriptive statistics and group difference analyses between those who worsened and those who improved in depression levels from baseline to follow-up.

	Worsened Depression *n* = 87	Improved Depression *n* = 121			
Demographics	*M* (*SD*) or Count (%)		*df*	*Statistic*	*p*
Age	36.6 (8.9)	34.6 (9.3)	206	*t* = −1.52	0.131
Gender			1	χ^2^ = 1.53	0.217
Male	54 (62.1%)	85 (70.2%)			
Female	33 (37.9%)	36 (29.8%)			
Race/Ethnicity			3	χ^2^ = 1.16	0.763
White, non-Hispanic	48 (55.2%)	58 (47.9%)			
Hispanic	16 (18.4%)	26 (21.5%)			
Black, non-Hispanic	14 (16.1%)	24 (19.8%)			
Other, non-Hispanic	9 (10.3%)	13 (10.7%)			
Education			1	χ^2^ = 0.03	0.875
Less than HS	57 (65.5%)	78 (64.5%)			
HS or greater	30 (34.5%)	43 (35.5%)			
Length of Incarceration			1	χ^2^ = 0.11	0.745
Six months or less	40 (46.0%)	52 (43.7%)			
More than six months	47 (54.0%)	67 (56.3%)			
Income at three weeks			1	χ^2^ = 9.35	0.002
Less than $20 K/year	76 (92.7%)	89 (76.1%)			
Living Situation at three weeks			1	χ^2^ = 2.42	0.120
Stable	57 (70.4%)	92 (80.8%)			
Unstable	24 (29.6%)	23 (20.0%)			
Physical health					
Health status at three weeks			1	χ^2^ = 4.87	0.027
Poor/fair/good	22 (56.4%)	25 (34.7%)			
Very good/excellent	17 (43.6%)	47 (65.3%)			
Stress					
PSS score at baseline	23.37 (6.31)	20.83 (5.89)	206	*t* = −2.97	0.003
PSS score at three weeks	21.36 (4.27)	14.88 (4.91)	206	*t* = −9.91	<0.001
Change in PSS score	−2.01 (6.78)	−5.96 (7.44)	206	*t* = −3.92	<0.001
Stress level at baseline			2	χ^2^ = 10.98	0.004
None (0–13)	5 (5.7%)	9 (7.4%)			
Mild (14–19)	12 (13.8%)	40 (33.1%)			
Severe (20+)	70 (80.5%)	72 (59.5%)			
Stress level at three weeks			2	χ^2^ = 81.25	<0.001
None (0–13)	3 (3.4%)	42 (34.7%)			
Mild (14–19)	16 (18.4%)	59 (48.8%)			
Severe (20+)	68 (78.2%)	20 (16.5%)			

*n* = 208; PSS = Perceived Stress.

**Table 2:** Descriptive statistics and group difference analyses between those who worsened and those who improved in stress levels from baseline to follow-up.

	Worsened Stress *n* = 88	Improved Stress *n* = 120			
Demographics	*M* (*SD*) or Count (%)		*df*	*Statistic*	*p*
Age	34.8 (8.9)	35.9 (9.4)	206	*t* = 0.88	0.379
Gender			1	χ^2^ = 1.29	0.256
Male	55 (62.5%)	84 (70.0%)			
Female	33 (37.5%)	36 (30.0%)			
Race/Ethnicity			3	χ^2^ = 5.99	0.112
White, non-Hispanic	49 (55.7%)	57 (47.5%)			
Hispanic	21 (23.9%)	21 (17.5%)			
Black, non-Hispanic	10 (11.4%)	28 (23.3%)			
Other, non-Hispanic	8 (9.1%)	14 (11.7%)			
Education			1	χ^2^ = 0.31	0.579
Less than HS	59 (67.0%)	76 (63.3%)			
HS or greater	29 (33.0%)	44 (36.7%)			
Length of Incarceration			1	χ^2^ = 0.59	0.444
Six months or less	42 (47.7%)	50 (42.4%)			
More than six months	46 (52.3%)	68 (57.6%)			
Income at three weeks			1	χ^2^ = 2.97	0.085
Less than $20 K/year	64 (72.7%)	84 (70.0%)			
Living Situation at three weeks			1	χ^2^ = 5.37	0.020
Stable	57 (67.9%)	92 (82.1%)			
Unstable	27 (32.1%)	20 (17.9%)			
Physical health					
Health status at three weeks			1	χ^2^ = 0.87	0.352
Poor/fair/good	21 (47.7%)	26 (38.8%)			
Very good/excellent	23 (52.3%)	41 (61.2%)			
Depression					
CES-D-10 score at baseline	14.64 (6.60)	10.71 (5.56)	168 *	*t* = −4.53	<0.001
CES-D-10 score at three weeks	13.49 (4.91)	5.82 (4.30)	206	*t* = −11.97	<0.001
Change in CES	−1.15 (6.83)	−4.89 (6.36)	206	*t* = −4.06	0.001
Depression level at baseline			2	χ^2^ = 13.91	0.001
None (0–9)	21 (23.9%)	54 (45.0%)			
Mild (10–14)	27 (30.7%)	38 (31.7%)			
Severe (15+)	40 (45.5%)	28 (23.3%)			
Depression level at three weeks			2	χ^2^ = 79.65	<0.001
None (0–9)	20 (22.7%)	101 (84.2%)			
Mild (10–14)	33 (37.5%)	12 (10.0%)			
Severe (15+)	35 (39.8%)	7 (5.8%)			

*n* = 208; CES-D-10 = 10-item Center for Epidemiologic Studies Depression Scale; * *t*-test with unequal variance used.

## 4. Discussion

This study examined changes in levels of depression and stress among prison inmates during and after incarceration in the U.S. Consistent with our hypothesis, we found that the majority of participant’s level of depression and/or stress improved post-release. However, there was a sizable minority of inmates who had an increase in symptoms (30.8% for depression and 29.8% for stress). This finding suggests that environmental stressors may be worse in the community than in prison or that post-release reintegration may be particularly challenging for some inmates.

Several studies have noted that for some inmates physical and psychological health may be positively influenced by being in prison due to the structural systems in place that provide inmates with consistent access to shelter, food, exercise, education and health care [18,19,20]. Furthermore, it has been suggested that for some inmates prison may serve as a refuge from the high rates of substance use or violence found within their communities. It has also been argued that incarceration may be a protective factor against mortality for some inmates whose life expectancy may be shorter in their communities due to detrimental environmental factors in comparison to the conditions provided within some prison facilities [21].

In the general U.S. population, the average CES-D-10 and PSS scores are 7–9 and 13–14, respectively [15,22]. Our sample reported much higher levels of depression and stress both before and after release from prison than non-incarcerated persons. The heightened depression and stress levels after release from prison may be associated with initial difficulties during the reintegration process, such as a lack of social support, financial issues, and poor coping styles [23]. An Australian study by Shinkfield and Graffam (2009) on the variables influencing successful reintegration after prison found that mean ratings of psychological health were significantly lower at one to four weeks after release than at pre-release and three to four months post-release among their participants, which may have been associated with unmet expectations or greater problems than anticipated after prison release. Financial issues may have also contributed to feelings of depression and stress, especially since participants in the Australian study tended to be underemployed at one to four weeks after release and employed only 33% of the days worked by a typical worker [24].

Our findings suggest that interventions need to be developed and tested that not only address depression and stress levels while inmates are still incarcerated but also need to occur immediately following incarceration in order to attend to changes in depression and stress levels post-incarceration, especially for those individuals who get worse. Meditation-based interventions (e.g., transcendental meditation, mindfulness-based stress reduction, and 10-day Vipassana retreats) have been shown to be effective in enhancing psychological well-being and decreasing substance use and recidivism among correctional populations [25]. It is quite possible that mindfulness/meditation-based techniques taught to inmates prior to release and then again as a booster follow-up session post-release may help to reduce their depression and stress levels during and after incarceration which may in turn assist them during the re-integration process and to prevent repeat incarcerations.

The current study has some limitations that are important to highlight. First, although our sample included both men and women, our findings may not generalize to all incarcerated populations in the rest of the U.S. or internationally. Second, the results are based on cross-sectional data and suggestions to intervene on depression or stress aimed at enhancing mental health problems would need to be examined longitudinally. Third, we examined depression and stress levels in the current study but we did not investigate specific mental health problems, such as major depressive disorder or post-traumatic stress disorder. Future studies should consider looking at potential changes in specific mental health concerns among inmates before and after being released from prison or jail. Fourth, we did not control for mental health treatment that inmates may have been receiving while incarcerated or afterwards. Finally, the time period in which we assessed levels of depression and stress (eight weeks prior to and three weeks after release from prison) may have impacted our findings. Different time points (e.g., three, six, or twelve months) in which levels of depression and stress are measured is important for future research to assess. It is quite possible that depression and stress levels may change the longer an individual is in or out of prison or jail.

## 5. Conclusions

Although this study has limitations, its major strength is that it is the first study to our knowledge to examine changes in depression and stress levels during and after incarceration in the U.S. This is particularly important given that a recent systematic review that provides prevalence estimates of psychiatric disorders from inmates in 19 U.S. state prisons found that as many as 29% and 49% of inmates suffer from major depression and posttraumatic stress disorder, respectively [26]. Future research is needed to address depression and stress levels among inmates for a healthier transition back into the community and to prevent re-incarcerations.
